# Plasmapheresis, Rituximab, and Ceftriaxone Provided Lasting Improvement for a 27-Year-Old Adult Male with Pediatric Autoimmune Neuropsychiatric Disorders Associated with Streptococcal Infections (PANDAS)

**DOI:** 10.1155/2021/8697902

**Published:** 2021-11-02

**Authors:** Adam Krouse, Huihua Li, Joseph A. Krenzer, William Nicholas Rose

**Affiliations:** Department of Pathology, University of Wisconsin Hospital, 600 Highland Ave, Madison, WI 53792, USA

## Abstract

Pediatric autoimmune neuropsychiatric disorders associated with streptococcal infections (PANDAS) is a specific autoimmune response to group-A streptococcal (GAS) infections in children and adolescents with a sudden onset of neuropsychiatric disorders including obsessive-compulsive disorder (OCD) or tic-like symptoms. We present a case report of a 27-year-old male patient who had lasting improvement with plasmapheresis, rituximab, and ceftriaxone. Our patient first developed sudden psychosis and confusion after GAS infections at age 17. He had elevated anti-streptolysin O (ASO) titers, negative urine drug screen, no ETOH in blood, normal CBC, normal TSH, normal salicylate, normal acetaminophen, and a normal head CT. The tentative diagnosis of PANDAS was made, and the patient was thereafter treated with antipsychotics, antibiotics, tonsillectomy, and IVIG which resulted in remissions and relapses of his neuropsychiatric symptoms. Once he reached age 27, he received a trial of therapeutic plasma exchange (TPE), rituximab, and ceftriaxone. This eventually resulted in sustained benefit and minimal fluctuations of his clinical symptoms. Our report is noteworthy in three ways.One, he is a 27-year-old adult with PANDAS.Two, he improved after TPE, rituximab, and ceftriaxone. Our literature search yielded minimal data on the use of plasmapheresis for nonteenage adults with PANDAS. Three, he had unusual symptoms of PANDAS, as the typical OCD and/or tic-like symptoms were not observed.

## 1. Introduction

Pediatric autoimmune neuropsychiatric disorders associated with streptococcal infections (PANDAS) was first described by Swedo et al. in the late 1990s and refers to a disease state characterized by the abrupt onset of neuropsychiatric symptoms. The most common are obsessive-compulsive disorder (OCD) and/or tics. PANDAS is a subtype of pediatric acute-onset neuropsychiatric syndrome (PANS) but with a temporal relationship to infection with beta hemolytic group-A streptococcal (GAS) infection.

Additional common symptoms that have been reported in patients within this diagnostic category include hyperactivity, inattention, psychosis, separation anxiety, behavioral regression, nighttime fears and rituals, decline in school performance, sensory issues, and oppositional behaviors [1]. PANDAS appears to have a male predominance [1].

Although the incidence and prevalence of PANDAS are not known, it is generally considered a rare disease [2]. However, some researchers suggest that it may account for ≥10% of childhood-onset obsessive-compulsive disorder (OCD) and tic disorders [2].

The exact etiology of PANDAS is not well-established. Some strong evidence exists for the role of infection in triggering the symptoms of tics and OCD observed in these patients. ASO titer levels correlate with OCD and tic severity [1].

In addition, PANDAS may also have an autoimmune component. Immunologic aberrations have often been observed in PANDAS patients that include findings similar to those seen in Sydenham's chorea (SC), such as evidence of GAS-mediated anti-neuronal autoantibodies, dopamine receptor autoantibodies, and basal ganglia pathology [1].

Given the relatively obscure pathophysiology and variable manifestations, an uncontroversial standard of care does not exist. Selective serotonin reuptake inhibitors (SSRIs) and cognitive behavioral therapy (CBT) are considered first-line therapy for acute-onset OCD spectrum and have been successful in patients with PANDAS [3]. The use of antibiotics is another commonly reported treatment strategy [4–6]. Immunomodulation therapies such as IVIG and plasmapheresis have also been used with some degree of reported success. Plasmapheresis, or therapeutic plasma exchange (TPE), has been shown to be effective in cases of severely ill patients, as demonstrated most recently by Latimer et al. in 2015 [7]. Many patients undergo multiple treatment modalities that are often administered in conjunction with one another and with several modifications during their clinical course in an effort to achieve symptom improvement.

## 2. Case Presentation

We present a 27-year-old-male with PANDAS who received therapeutic plasma exchange (TPE), rituximab, and ceftriaxone with significant improvement. His medical history is notable for asthma, marijuana use, and family history of schizophrenia. Psychiatric family history includes a maternal great grandmother with schizophrenia and a paternal great aunt with an unidentified mental illness requiring institutionalization. About 10 years prior to TPE treatment, the patient was brought to the emergency room by family members for newly developed paranoia and confusion. One month prior to the ER presentation, he had a sore throat, muscle weakness, and fever. His monospot and strep culture were negative at that time. He was treated for presumed mononucleosis.

In the ER, the patient endorsed insomnia, confusion, and paranoia with persecutory delusions. He denied hallucinations or suicidal ideations. Evaluation at that time revealed a negative urine drug screen, no alcohol in his blood, normal complete blood count, normal thyroid-stimulating hormone, normal salicylate, normal acetaminophen, and a normal head computed tomography (CT). His urinalysis was only remarkable for moderate hemoglobin and >50 erythrocytes. Physical exam was only positive for a cardiac murmur and for appearing generally distressed. He reported intermittent alcohol and marijuana use but nothing associated with this acute presentation, and this was supported by the negative laboratory tests. He reported that his last uses of marijuana and alcohol were 2-3 days prior at a party.

Psychiatry was consulted. At the time of their evaluation, the patient denied any delusions or confusion. Mental status exam at the time revealed normal orientation, anxious mood, mildly restricted affect, and a somewhat tangential and disorganized thought process. His judgement and insight had improved from prior. His family reported that for a few days prior to presentation, the patient had been making some paranoid comments such as believing his water was contaminated. However, these symptoms fluctuated. He was diagnosed with an acute stress reaction, prescribed trazodone to help him sleep, and told to follow up with psychiatry as an outpatient. The next day, the patient's symptoms returned, and his family was advised to take him to the ER again. He was then admitted to an inpatient adolescent psychiatric unit. An anti-streptolysin O (ASO) antibody test was performed and was elevated at 835 with a reference range of 0-330. He was discharged from the inpatient unit with a tentative diagnosis of PANDAS and was started on aripiprazole, lorazepam, and azithromycin.

Due to side effects, aripiprazole and lorazepam were discontinued. He received further antibiotic treatment with cefdinir and reported improvement which lasted approximately 2 years until he relapsed.

His symptoms of impaired concentration and slowed cognitive processing continued to wax and wane with intermittent treatment of antibiotics and antipsychotics. The symptoms were of such a magnitude that they impaired his ability to work, thus prompting the patient to start intermittent IVIG therapy. He received relief with IVIG therapies but would subsequently relapse.

Additional workup during this time included a head MRI which was normal. A lumbar tap showed a glucose of 79 and a protein of 27. He also had a mild elevation of GAD65, a negative anti-NMDA receptor test, an electroencephalogram only notable for beta activity, and a paraneoplastic battery which was normal.

He was treated with additional IVIG with an induction dose of 2 g/kg and then 0.5 g/kg weekly for maintenance. His maintenance dose was increased to 1 g/kg due to symptom recurrence. He reported significant relief of symptoms at this time. The maintenance dose was spaced out to every 2 weeks and then every month along with the addition of 1,000 mg BID mycophenolate and 30 mg prednisone.

He did relatively well during this time, but he reported fluctuations in his neuropsychiatric symptoms, specifically his ability to concentrate and his cognitive function. He reported that his ability to work was hampered by the decreased cognitive function he felt during these fluctuations. Given the several previous therapies that did not provide lasting improvement along with sparse case reports that plasmapheresis may help with a minimal side effect profile, it was decided to give the patient a trial of TPE.

The IVIG was stopped, his mycophenolate 1000 mg BID was continued, and the prednisone was tapered down to 10 mg a day. TPE was performed 5 times over 11 days on a Monday, Wednesday, Friday (MWF) schedule. The plasmapheresis procedures were all done using centrifugal apheresis machines using 1-plasma volume exchanges with 5% albumin as the replacement fluid. The TPEs were tapered to every 2 weeks. He tolerated the TPEs well.

Due to return of symptoms along with migraine, nausea, and dizziness, the patient again received a series of 5 TPEs over 11 days on a MWF schedule. Patient then transitioned to weekly TPE. He reported that he would feel symptoms return 2-3 days prior to next TPE.

The decision was made to add rituximab to his treatment regimen. He continued weekly TPE and transitioned to every 2 weeks and then every 3 weeks. In total, he received 35 TPEs over 11 months. He received 3 infusions of rituximab; the first 2 were 2 weeks apart, and the last one was 6 months later. He received 500 mg IM ceftriaxone weekly which was tapered to every 3 weeks. With the rituximab, TPE, and ceftriaxone, the patient ultimately reported sustained benefit and minimal fluctuations of his symptoms after cessation of the above 3 therapies.

## 3. Discussion

### 3.1. Diagnosis

The 5 diagnostic criteria proposed for PANDAS include (1) presence of obsessive-compulsive disorder (OCD) and/or a tic disorder, (2) prepubertal onset, (3) abrupt onset or exacerbation of symptoms with an episodic (relapsing-remitting) course, (4) temporal association of symptoms with GAS infection, and (5) association with neurological abnormalities including choreiform movements [4]. Although the peak age of onset for PANDAS is 6-7 years, PANDAS has also been reported in adults [8]. There are no specific confirmatory laboratory tests. This makes the diagnosis of PANDAS clinically challenging and, to some physicians, controversial.

Positive throat culture result and/or an elevated anti-streptococcal antibody titer following GAS infection support the diagnosis, but many experts claim they are not always present in PANDAS patients. Some authors consider PANDAS to be an autoimmune process, as elevated levels of anti-neuronal antibodies and/or anti-basal ganglia antibodies have been reported [9]. In addition, magnetic resonance imaging (MRI) studies have demonstrated enlargements of the caudate, putamen, and globus pallidus [10]. And, in some patients, the sizes of basal ganglia structures were normalized following successful immunomodulatory therapy with IVIG or plasma exchange [11].

The clinical symptoms of PANDAS can be broad. In addition to OCD and tic-like symptoms, hyperactivity, inattention, psychosis, separation anxiety, behavioral regression, nighttime fears and rituals, decline in school performance, sensory issues, and oppositional behaviors all have been reported in patients who have been diagnosed with PANDAS [12, 13]. No anti-neuronal antibodies or anti-basal ganglia antibodies have been found in this patient. His head MRI also showed relatively normal findings.

We freely concede that, apart from elevated ASO antibody titers, this patient has no overtly objective findings that definitively prove that he has PANDAS. However, many patients that are labeled with PANDAS as a tentative or working diagnosis also lack many or even any objective findings. Within the relatively immature field of psychiatry (and to some degree, neurology), this is common. In other words, this is not unique to PANDAS, as many neuropsychiatric conditions such as many mood, behavioral, and psychotic disorders lack lab tests, imaging, or tissue findings that rule the condition in or out.

Perhaps most importantly, PANDAS itself lacks an uncontroversial definitive set of diagnostic criteria. At minimum, this patient could be said to have a PANDAS-like neuropsychiatric syndrome with a pathophysiology that seems to include ASO antibodies. It could be a separate condition from PANDAS, or splitting them into different labels may simply be a distinction without a difference. We cannot say (nor do we think that anyone in 2021 could say) with certainty.

### 3.2. Treatment

Currently, the treatment options for PANDAS include cognitive behavioral therapy and/or antiobsessional medications, antibiotics, tonsillectomy, IVIG, and plasmapheresis. Antibiotic treatment is often indicated in patients with PANDAS with tonsillopharyngitis and a positive GAS throat culture. In a double-blind randomized controlled trial, penicillin and azithromycin prophylaxis was found to be effective in decreasing streptococcal infections and neuropsychiatric symptom exacerbations in children with PANDAS [4, 5].

Surgical interventions, including tonsillectomy and/or adenoidectomy, have been considered in children with PANDAS, although one study has showed that tonsillectomies and adenoidectomies did not prevent the onset of PANDAS [14]. In severely symptomatic patients with PANDAS, immunomodulatory therapies including intravenous immunoglobulin (IVIG) or TPE may be effective in reducing symptom severity or shortening disease course [4, 15].

In addition, Perlmutter et al. reported that plasma exchange and IVIG seemed to be effective in lessening of symptom severity for children with infection-triggered OCD and tic disorders [11]. Latimer et al. studied TPE treatment in 35 severely ill children and adolescents with PANDAS, and they found that all patients appeared to have had at least some benefit with average improvement of 65% at 6-month post-TPE and 78% at longer-term follow-up [7]. TPE as a potentially effective therapy for PANDAS has also been reported from in other case reports [16–18].

In the present case, the patient was initially treated with antipsychotics and antibiotics including azithromycin and cefdinir following the diagnosis of PANDAS. Due to the relapse of the neuropsychiatric symptoms, he then was treated with IVIG and bilateral tonsillectomy followed by TPE, rituximab, and ceftriaxone. Although TPE has been reported as a potential therapy in children and adolescents with PANDAS, the data on TPE for adults with PANDAS is extremely sparse.

His initial doses of TPE resulted in temporary relief and fluctuations of symptoms. With the combination of TPE, rituximab, and ceftriaxone, he eventually achieved sustained benefit and minimal fluctuations of his symptoms. To our knowledge, this specific treatment regimen has not been reported previously with a successful outcome for an adult with PANDAS.

## 4. Conclusion

We share our experience with a patient with sudden psychosis and confusion after GAS infections who was eventually treated at age 27 with the combination of TPE, rituximab, and ceftriaxone that resulted in sustained benefit and minimal fluctuations of his symptoms that has lasted after the cessation of treatment. The combination of immunomodulatory therapy (TPE and rituximab) with antibiotics (ceftriaxone) appeared to have worked well in controlling the symptoms in this adult patient with PANDAS. While our data is meager, we offer our report of this patient in case other physicians and patients find it useful.

## Data Availability

No data were used to support the findings of this study.

## Abbreviations

TPE: Therapeutic plasma exchange
PANDAS: Pediatric autoimmune neuropsychiatric disorders associated with streptococcal infections
OCD: Obsessive-compulsive disorder
ASO: Anti-streptolysin O
IVIG: Intravenous immunoglobulin
GAS: Group-A streptococcus.
